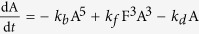# Adapting to Adaptations: Behavioural Strategies that are Robust to Mutations and Other Organisational-Transformations

**DOI:** 10.1038/srep18963

**Published:** 2016-01-08

**Authors:** Matthew D. Egbert, Juan Pérez-Mercader

**Affiliations:** 1 Harvard University, Dept. of Earth and Planetary Sciences, Cambridge, 02142, USA; 2 Santa Fe Institute, Santa Fe, 87501, USA

## Abstract

Genetic mutations, infection by parasites or symbionts, and other events can transform the way that an organism’s internal state changes in response to a given environment. We use a minimalistic computational model to support an argument that by behaving “interoceptively,” i.e. responding to internal state rather than to the environment, organisms can be robust to these *organisational-transformations*. We suggest that the robustness of interoceptive behaviour is due, in part, to the asymmetrical relationship between an organism and its environment, where the latter more substantially influences the former than vice versa. This relationship means that interoceptive behaviour can respond to the environment, the internal state and the interaction between the two, while exteroceptive behaviour can only respond to the environment. We discuss the possibilities that (i) interoceptive behaviour may play an important role of facilitating adaptive evolution (especially in the early evolution of primitive life) and (ii) interoceptive mechanisms could prove useful in efforts to create more robust synthetic life-forms.

The regulatory processes performed by organisms and proto-organisms can be divided into two main categories. *Interoceptive* regulation is driven by a response to an organism’s internal-state. A good example of this is ‘end-product inhibition’ where a metabolic pathway is inhibited by the presence of its product (see e.g.^1^). In contrast, when regulation is driven by a response to environmental cues, such as the induction of enzyme synthesis by the presence of certain reactants in the local environment of the organism (see e.g.^2^) we call this *exteroceptive* regulation. We tend to think of regulation as an operation upon internal processes, but it is also common for organisms to regulate their interaction with their environment. Motile organisms change their local environment by relocating, and a wide variety of organisms modulate their enviroment interface (e.g. single-celled organisms can regulate the permeability of their membrane) thereby changing how they interact with their environment. The regulation by an organism of its interaction with its environment corresponds to a wide class of dynamics that we (following^3^,^4^) broadly refer to as *behaviour*. Behaviour is predominantly seen as exteroceptive —driven by a response to environmental cues (e.g.^5^,^6^,^7^), but there are examples of interoceptive behaviour, where for instance, movement is driven by a response to the internal-state of the organism —see, for instance, the metabolism-dependent chemotaxis of several species of bacteria^8^,^9^,^10^,^11^.

Events such as genetic mutations, infection by parasites or symbionts, and chance environmental encounters can modify the way that an organism’s internal state changes in response to a given environment. Some of these *organisational-transformations* provide a fitness advantage, but the majority are deleterious. In some cases, the disadvantageous effects of these events could be reduced or neutralised *if* there were a coincident adaptation in organism’s regulatory processes that compensated for the change. Similarly, there are also organisational-transformations that would provide an advantage, but *only if* there were a coincident change in the behaviour of the organism. As an example of this, consider a mutation that causes an organism to be able to metabolise a new type of resource. This mutation would only be beneficial if the behaviour “adapted to the adaptation,” — i.e. changed so that the organism started to consume that resource. An organism that automatically adapted its behaviour to accommodate these kinds of changes would have fewer deleterious and more neutral or beneficial organisational-transformations than a system that does not. It would therefore more rapidly accumulate neutral and beneficial mutations, and in this way would be a more evolvable organism. We can then ask: is there a mechanism or a form of regulation that automatically adapts to the kinds of changes brought about by mutations and other organisational-transformations?

In most cases, neo-Darwinian evolution would be too slow to produce the required adaptation as either (a) the organisational-transformation is not heritable and there would be no chance for natural selection to operate, or (b) even in the cases where the organisational-transformation is heritable, most are neutral or deleterious and so natural selection or genetic drift would likely eliminate them^12^ in a small number of generations^13^, before the adaptation in regulation could take place.

In this paper, we argue that even extremely simple forms of interoceptive regulation are naturally suited to accomplishing this kind of adaptation. We argue that interoceptive behaviour (unlike exteroceptive behaviour) can automatically adapt to organisational-transformations, regulating the local environment or internal processes so that the organism remains viable despite potentially radical changes in its needs or capabilities. In being robust to mutations and other heritable organisational-transformations (such as some forms of symbiosis), interoceptive behaviour could have played an important role facilitating adaptive evolution, especially that of early forms of life, where organisational-transformations were likely more frequent and radical. We further suggest that the robustness of synthetic protocells could be improved by the implementation of interoceptive forms of behaviour where, for instance, membrane permeability or motility mechanisms are operated in response not to the environment of the protocell, but to its internal state and needs. We mention in the discussion some experimental work that has already started to move in this direction.

The remainder of this paper proceeds as follows: we first present the mathematical framework that we use to formally define and investigate the robustness of interoceptive and exteroceptive behaviour in response to organisational-transformations. An argument is then made as to why we might expect interoceptive behaviour to be more robust to organisational-transformations than exteroceptive behaviour. We then present an abstract model that is used to perform a systematic survey of organisational-transformations, where we evaluate their impact upon interoceptive and exteroceptive forms of behaviour. As predicted, the interoceptive behaviours examined in the model prove to be more robust than the exteroceptive behaviour. In the discussion, we elaborate as to why interoceptive behaviour demonstrates a natural robustness to these kinds of events, unlike exteroceptive behaviour which tends to be quite fragile by comparison. We close by explaining how the results of our analysis are relevant to evolution and synthetic life.

## Methods

### Interoceptive and exteroceptive behaviour

Following Ashby’s framework for the study of adaptivity^3^, we will define the *internal state* of an organism, *I*, as the state of a set of “essential variables,” i.e. variables that must remain within limits if the organism is to be considered alive. We then define the *local environment* of an organism, *E*, as the subset of the global environment, *G*, that influences the internal state of the organism, and assume that the internal state changes as a function, *f*, of its current state and the state of its local environment:


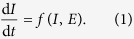


The local environment, *E*, is influenced by the global environment. For instance, seasonal change causes a bear’s local environment to change temperature. But the local environment also changes as a result of the organism’s behaviour —the bear can seek shelter, mitigating, neutralising, or overwhelming the effects of seasonal change. We can thus write that the local environment of an organism changes as a function of the global environment, *G*, and behaviour, *B*:


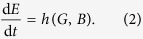


We consider two forms of behaviour. The first is an exteroceptive response to the organism’s environment. We can describe the effect of an exteroceptive behaviour as a function of its global environment. Substituting the function x(G) for *B* in Equation (2), we get:


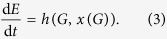


The second form of behaviour is an interoceptive response to the internal state of the organism, i.e. to one or more essential variables. Note that our usage of “exteroceptive” and “interoceptive” distinguishes between phenomena by how they relate to the viability of the organism, not by their physical location; interoceptive behaviour is a response to an essential variable and exteroceptive behaviour is a response to anything else. Substituting the function n(I) for *B* in Equation (2), for the interoceptive behaviour we get:


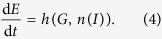


It is also possible for behaviour to respond to both *I* and *G*, but for simplicity we do not address that case at the moment. Coupling Equation (1) with Equations (3) and (4), we have two systems of coupled differential equations:

*System 1: “Exteroceptive”*


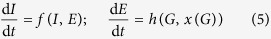


*System 2: “Interoceptive”*


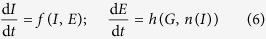


We use these systems and variations of them, to investigate the robustness of exteroceptive and interoceptive behaviour to *organisational-transformations*.

### Organisational-transformations

An organisational-transformation is an event that modifies how essential variables change in a given environment (how *I* changes for a given *E*). We can formalise this as the transformation of the “pre-transformation” function *f* into a “post-transformation” form, *f* ′, defining two new systems of equations:

*System 1b:* “*Post-transformation Exteroceptive”*


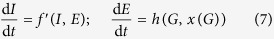


*System 2b: “Post-transformation Interoceptive”*


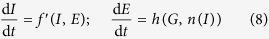


Genetic mutation has been given as an example of an organisational-transformation, but this is not the only phenomenon that this formalisation captures. The notion encompasses a wide variety of events, including some that cause long-term or permanent change to the organism, or to the way that an environment affects the organism. Examples include:

1. genetic mutations that permanently increase or decrease the rate at which an organism can metabolise a resource, or that modify an offspring’s ability to metabolise a whole category of resources;
2. the infection of an organism by a parasite or symbiont, causing it to require additional resources to survive, or alternatively, allowing it to benefit from a new category of resources;
3. an event that has long-term implications, such as a rare encounter with a chemical that permanently modifies the organism’s metabolism; or
4. damage to an organism (e.g. membrane damage causing a slow but steady loss of metabolic products).

These kinds of events are common at evolutionary time scales, and it would clearly be advantageous to be robust to them, i.e. to remain healthy despite them.

### Behavioural robustness to organisational-transformations

**Figure 1:**
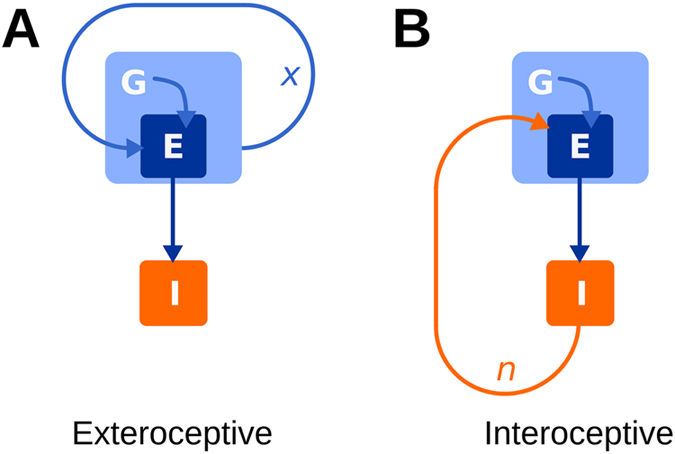
Influence in the two systems defined by Equations (5) and (6). In the exteroceptive system (**A**), the local-environment, *E*, is regulated in response to the global environment, *G*. In the interoceptive system (**B**), *E* is regulated in response to essential-variables, *I*.

Why might we expect interoceptive behaviour to be more robust to organisational-transformations than exteroceptive behaviour? Organisational-transformations modify how the internal-state changes in a given environment. It follows that in general, an ideal environment for maintaining viability *pre-transformation*, will not be ideal *post-transformation*, and a change is therefore required in the organism’s behaviour if it is to optimally regulate its interaction with its environment. Exteroceptive behaviour will not produce any such change. We can say this because organisational-transformations modify the dynamics of internal-state variables, but by definition, exteroceptive behaviour is not influenced by these variables (see Fig. 1A and Equation (5)) and so post-transformation exteroceptive regulation will be no different than pre-transformation exteroceptive regulation. Put another way, the environmental needs of the system have changed, but purely exteroceptive behaviour will continue to regulate environmental conditions in the same way that it did before the organisational-transformation.

Interoceptive behaviour is different. It closes a sensorimotor loop that includes both environmental and internal-state dynamics (see Fig. 1B and Equation (6)). Instead of being decoupled from the internal state, interoceptive behaviour is a direct response to it. An organisational-transformation changes the way that an environment affects the internal state and when behaviour is interoceptive, the different internal state will result in a different regulation of the local environment. After organisational-transformations the “right” environment will likely have changed, but the right internal state remains the same. We thus might expect interoceptive behaviour to be more robust to organisational-transformations than exteroceptive behaviour.

## Results

We now use an abstract model to evaluate the propositions of the previous section. The model acts as an instantiation of the concepts described above, simulating the dynamics of an organism’s internal state that is affected by its local environment and where the interface between the two is regulated either interoceptively or exteroceptively. We conduct a systematic survey, subjecting interoceptive and exteroceptive behaviours to a range of parameter-modification based organisational-transformations. As predicted above, the exteroceptive behaviour turns out to be much less robust to organisational-transformations than the interoceptive behaviours.

### Model of Metabolism

Our minimal model simulates the dynamics of the concentration of two distinct categories of chemical. ‘A’ is an internal state variable (i.e. A ∈ *I*) which represents the concentration of all of the different chemicals that constitute an organism’s autocatalytic metabolic network. ‘F’ is an environmental variable (F ∈ *E*) which represents the concentration of resources present in the environment of the organism that can be transformed by the metabolic-network into metabolites.

The metabolism grows and degrades according to the two following reactions:


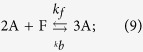



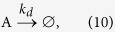


where Reaction (9) represents the autocatalytic transformation of F into A, and Equation (10) captures the degradation of A into something that has no subsequent influence on the system. Applying mass-action kinetics, we get


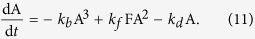


which defines the pre-transformation internal state dynamics (comparable to function *f* in Equations (5) and (6)) in our example system.

We arbitrarily define limits on this essential variable, saying that A must lie between 0 and 8 if the system is to be considered viable. These viability limits, and the other constants (the stoichiometry of the reaction and its rate-constants *k*_*b*_ = 0.075, *k*_*f*_ = 0.5, *k*_*d*_ = 1) were selected to produce a system in which certain initial conditions survive and others do not, and where regulation of the environmental conditions (i.e. behaviour) can influence which initial conditions survive and which do not. In our analysis below, we systematically modify the stoichiometric and rate-constant parameters, comparing the ability of interoceptive and exteroceptive behaviours to drive survival-prolonging behaviour. As predicted by the reasoning presented above, the interoceptive behaviours demonstrate a greater resilience to these modifications than the exteroceptive behaviour.

**Figure 2:**
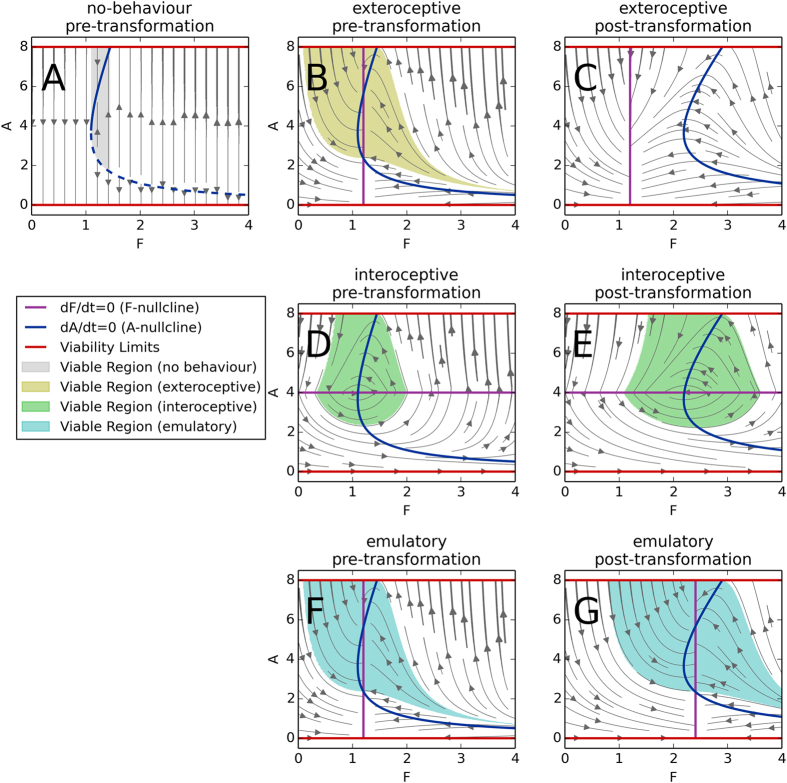
Dynamics and viable regions in pre-transformation and post-transformation conditions. The first column indicates the system with no behaviour (fixed F). The second column indicates the pre-transformation dynamics of the three behaviours, and the third column shows the dynamics of the same behaviours after an example organisational-transformation, where *k*_*f*_ has been reduced by 50%. In each plot, the viability limits are indicated by the red lines, nullclines are indicated by the blue and purple curves, the viable region (initial conditions that do not encounter the viability limits) is shaded, and dynamics are indicated by the grey arrows.

Figure 2A shows A-dynamics for different fixed values of F. In between the viability limits (red lines) lies a set of equilibria (blue curve). For states that are to the right of this curve, A is increasing, and to the left of the curve, A is decreasing (grey arrows). We see that when there is a low concentration of F, the system is incapable of growing at a rate sufficient to compensate for its degradation, resulting in a single stable ‘dead’ equilibrium of no autocatalyst (*A* = 0). For a range of fixed concentrations of 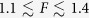, the system has two equilibria: a stable ‘living’ equilibrium, an unstable living equilibrium, and two ‘dead’ final states (*A* = 0, *A* = 8). And finally, for fixed 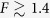, the system has three possible final states: the two dead states and an unstable equilibrium in between. In the plots in Fig. 2, the *viable regions* (i.e. the initial conditions that do not encounter the viability limits —see^14^) are shaded.

### Behaviours

For simplicity, our model omits the influence of the *global* environment (*G*) upon the local-environment and the behaviour. The model thus corresponds to scenarios where (i) the behaviour is sufficiently influential that it is not overridden by the influence of the global environment, and (ii) the effect of the behaviour upon the local-environment is independent of the non-local environment. The simplification allows us to describe change in the local environment variable F (analogous to the function *h* described above) as purely the result of the organism’s behaviour.

We investigate two interoceptive and one exteroceptive behaviours. The **minimal exteroceptive behaviour** is defined by:


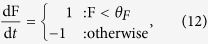


which causes F to increase if it is too low and decreases it if it is high (see Fig. 2B). The value of parameter *θ*_*F*_ = 1.2 was determined by approximating the middle of the viable region in the absence of behaviour (grey area in Fig. 2A). This equation is comparable to the function *x*(G) in the framework outlined above.

The **minimal interoceptive behaviour** is defined by the following equation (which plays the role of the function *n*(I)in the framework outlined above):


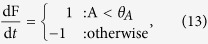


which causes F to increase when A is low and vice-versa, when A is high, F is reduced. The value of parameter *θ*_*A*_ = 4 was selected as it is precisely halfway between the viability limits (see Fig. 2A). This mechanism, like the minimal exteroceptive behaviour, succeeds at regulating F, such that the set of initial conditions that survive is larger than without behaviour (see Fig. 2D).

The two minimal behaviours just described perform qualitatively differently in the same pre-transformation conditions. To evaluate the impact of organisational-transformations upon interoceptive and exteroceptive behaviours that perform precisely the same regulation *pre-transformation*, we designed a third **emulatory behaviour**, which is an interoceptive behaviour that performs precisely the same as the minimal exteroceptive behaviour described above. To formulate this behaviour, we see from Equation (11) that:


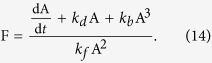


We can then imagine some (perhaps complicated) machinery that is not directly influenced by F but maintains the concentration of a chemical, *P*, as proportional to the right-hand-side of Equation (14), thus:


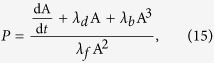


where the parameters *λ*_*f*_, *λ*_*b*_ and *λ*_*d*_ are given the same pre-transformation values as *k*_*f*_, *k*_*b*_, and *k*_*d*_ respectively. The interoceptive “emulatory” behaviour is then the same as the exteroceptive behaviour, except that it is a response to *P*:


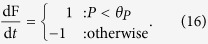


The value of the parameter *θ*_*P*_ has the same value as *θ*_*F*_ = 1.2 such that in pre-transformation conditions, this interoceptive behaviour performs precisely the same as the minimal exteroceptive behaviour (as confirmed by identical Figs. 2B,F).

### Organisational-transformations

To evaluate the robustness of these three systems, we systematically generated 9^3^ = 729 organisational-transformations by varying the rate-constants, *k*_*f*_, *k*_*b*_ and *k*_*d*_ in Equation (11) by the following scaling factors: 
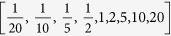
. (For more extreme parameter changes, the rate of change in F 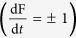 is often insufficiently fast to compensate for the organisational-transformation.) For each of these 9^3^ systems, for each of the three behaviours, we simulated trajectories from 16,384 initial-conditions distributed in a regular 128 × 128 lattice over A_*t*=0_ ∈ [0, 4], F_*t*=0_ ∈ [0, 8]. Each initial condition was considered to have survived if, after sufficient time for the system to come to rest (visually approximated to be 50 time units), 0.1 < A < 7.9.

**Figure 3:**
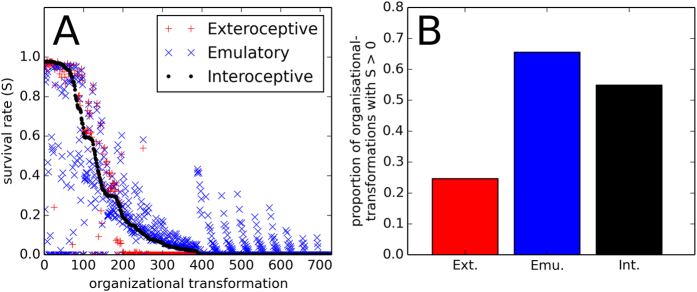
Robustness to systematic rate-constant modifications. The left plot indicates the survival rate (proportion of tested initial conditions that survive) for each of the three behaviours under each of the 729 organisational-transformations. To facilitate comparison of the behaviours, in this plot, the organisational-transformations (horizontal-axis) have been sorted according to the survival rate of the minimal interoceptive behaviour. The bar-chart shows the proportion of organisational-transformations that are survived by at least one initial condition for each behaviour. The two interoceptive behaviours are clearly more robust to the organisational-transformations than the exteroceptive behaviour.

Figure 3A plots the proportion of initial conditions that survive for each behaviour for each organisational-transformation. In a few cases, the exteroceptive behaviours out-performs the minimal interoceptive behaviour. This is not surprising, because these two behaviours are different even before the organisational-transformation. The more general result is that the two interoceptive behaviours are significantly more robust to organisational-transformations; their regulation drives survival in many conditions where the exteroceptive behaviour does not, as indicated in Fig. 3B.

The third column of Fig. 2 shows the post-transformation dynamics of the three behaviours after an example organisational-transformation, where *k*_*f*_ was reduced by 50%. As predicted, the exteroceptive regulation of F is not affected by the organisational-transformation —the 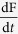 nullcline and more broadly the dynamics in F are identical in Plots B and C. Accordingly, F approaches 1.2, the same value it approached in the pre-transformation system. Unfortunately for the simulated organism, in the post-transformation system this environmental state inevitably leads to a loss of viability regardless of the initial condition. The exteroceptive behaviour is clearly not robust to the organisational-transformation.

The situation is quite different for the minimal interoceptive behaviour. The interoceptive regulation causes F to approach a higher value, and the post-transformation approached values of A are precisely the same as the pre-transformation values. Unlike for the exteroceptive behaviour, there is a large post-transformation viable region. The regulatory mechanism is simple, but the system has adapted to regulate the environmental conditions to produce the same internal state, despite the organisational-transformation.

In the emulatory behaviour, we see a similar result as in the minimal interoceptive behaviour, the post-transformation approached value of A (stable point indicated by the upper intersection of nullclines) is the same as in the pre-transformation system, and again there is a large viable region. Both interoceptive behaviours demonstrate a remarkable robustness to the organisational-transformation, unlike the exteroceptive behaviour.

**Figure 4:**
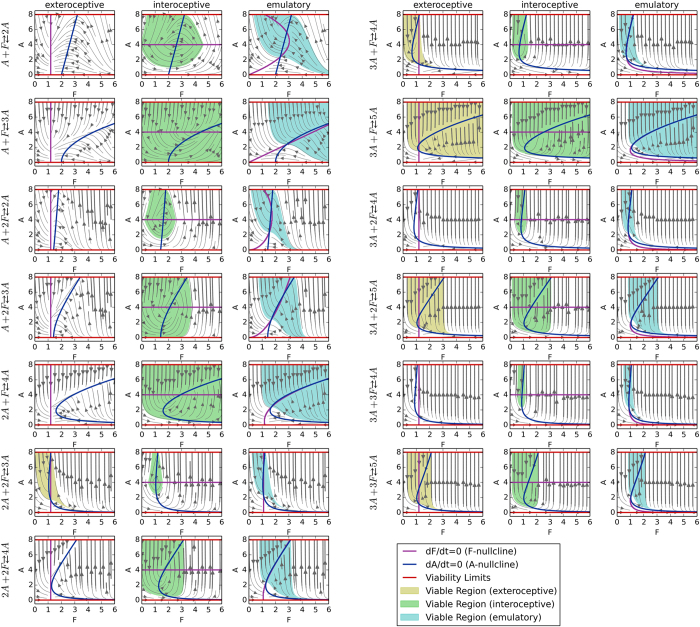
Robustness to stoichiometric modifications. These plots show the dynamics and viable regions for thirteen stoichiometry-based organisational-transformations. For each organisational-transformation, the dynamics and viable regions are indicated for each of the three behaviours. In many cases where the exteroceptive behaviour fails to maintain a viable region, the interoceptive behaviours succeed, demonstrating their greater robustness to the stoichiometric organisational-transformations.

To evaluate the effects of more profound organisational-transformations, we systematically modified the stoichiometry in Equation (9) (see Table 1). The stoichiometric number for each of the reactants was selected from {1, 2, 3} and the stoichiometric number for the product was selected such that *A* increases by either one or two. The three behaviours were simulated in the same way as above and the reaction rate constants (*k*_*f*_, *k*_*b*_ and *k*_*d*_) were also unchanged. In all of the thirteen tested stoichiometric organisational-transformations, the interoceptive and emulatory behaviours were capable of surviving some initial conditions (see green and blue regions in Fig. 4). In contrast, exteroceptive behaviour only maintained a viable region in five of the thirteen tested stoichiometric organisational-transformations.

## Discussion

Interoceptive behaviour, like other forms of phenotypic plasticity allow an organism to adapt to new environments. This flexibility makes it possible for organisms to survive for long enough such that neo-Darwinian evolution can operate. It is also now widely accepted that phenotypic plasticity can also facilitate adaptive evolution by influencing selection pressures via the Baldwin effect^15^,^16^,^17^,^18^. Interoceptive behaviour can provide these benefits, but as demonstrated in the model above, it can also automatically adapt to modifications in the way that an organism’s internal state changes in a given environment. Put another way, interoceptive behaviour can adapt to changes in the organisation of the organism itself^19^. When metabolic rate and stoichiometric constants were changed in our model, the interoceptive behaviours automatically adapted to the change, finding new environmental conditions that suit the needs of the modified organism. No additional mechanism of adaptation was necessary for the interoceptive behaviour(s) to survive despite a wide range of organisational-transformations.

To see how this automatic adaptation can facilitate adaptive evolution, we need only to remember that organisational-transformations include heritable modifications such as genetic mutations. Consider a mutation that provides a minor fitness advantage, but also changes the ideal operating conditions for the organism’s metabolism. Interoceptive behaviour (as demonstrated above) can automatically compensate for the changed needs, increasing the likelihood of the mutation becoming fixed in the population. In contrast, when behaviour is exteroceptive, organisational-transformations that change the optimal operating conditions will include a negative influence on fitness, for the exteroceptive behaviour will seek the same environment as that sought before the organisational-transformation took place. Interoceptive behaviour can “adapt to the adaptation,” making beneficial what would be a deleterious mutation if behaviour were purely exteroceptive.

What underlies this difference between interoceptive and exteroceptive behaviours? An organism’s internal state is influenced by its environment, but in the absence of interoceptive behaviour, the converse does not generally hold; i.e. the environment is *not* particularly influenced by the organism’s internal state. We say this because the environment of an organism is generally a larger system with greater “inertia” and with a greater number of factors influencing its trajectory than just a single organism within it. An organism, conversely, is influenced by little other than itself and its environment. This asymmetrical relationship means that the feedback loop closed by exteroceptive behaviour (see Fig. 1) only allows for a response to changes in the environment, where as the loop closed by interoceptive behaviour allows the system to respond to changes in the internal dynamics of the organism, changes in the environment, and to changes in how these two systems interact. In fact, there are some environmental changes that interoceptive behaviour would be better at adapting to than exteroceptive behaviour. For example, if a completely new type of metabolic resource emerged, exteroceptive behaviour would have to evolve a way of sensing that resource to be able to seek it out, but interoceptive behaviour would respond to it the way it responds to other resources^19^. Similarly, an environmental change that modifies the way that phenomena influence an organism (e.g. the transformation of a resource into a toxin by the presence of another reactant) is automatically adjusted for when behaviour is interoceptive^11^,^19^. Finally, it is interesting to note that when an interoceptive behaviour is present, it becomes possible for exteroceptive behaviours to respond indirectly to the internal state (via the interoceptive behaviours influence upon the environment). This is a topic for future study.

The advantages of interoceptive behaviour and its simplicity draw attention to the possibility of it playing an important role in facilitating the early evolution of life. Interestingly, some simple abiological chemistry, such as motile oil-droplets^20^,^21^,^22^,^23^, BZ-gel “worms”^24^, simulated reaction-diffusion “spots”^25^, simulated self-replicating domains^26^ and idealised protocells^27^ demonstrate what could be described as interoceptive behaviour —they move towards environments that enable or accelerate their metabolism-esque dissipative chemical reactions. Along similar lines, the advantages of interoceptive behaviour also suggest it as a candidate mechanism that could be of use in efforts to synthesise more robust, adaptable and sophisticated synthetic protocell systems^28^ and bio-reactors^29^ —systems that are currently only capable of operating in highly regulated lab conditions.

### Recapitulation

This paper started by distinguishing between two categories of regulation: *exteroceptive* regulation, which is a response to the environment and *interoceptive* regulation, which is a response to essential variables. We then defined *organisational-transformations* as events that modify how essential variables change in a given environment, and presented an argument as to why we would expect exteroceptive behaviour to be less robust to organisational-transformations than interoceptive behaviour. This argument stemmed from (i) the observation that exteroceptive behaviour will seek out the same environments post-transformation that it sought pre-transformation, but that for interoceptive behaviour, the organism can find different environments that may be better suited to its changed needs; and (ii) the identification of an asymmetrical causal relationship where (in the absence of interoceptive behaviour) an organism’s internal state is more influenced by its environment than vice versa. This asymmetrical relationship limits the sensitivity of exteroceptive behaviour, while allowing interoceptive behaviour to adapt not only to changes in the organism’s essential variables, but also to changes in its environment and to the interaction between the two.

As an example system that we could use to clarify and further evaluate our argument, we formulated a minimal abstract model, where we systematically subjected interoceptive and exteroceptive behaviours to a variety of organisational-transformations. As predicted, the minimal interoceptive behaviours demonstrated a much greater robustness to organisational-transformations than the exteroceptive behaviour. Obviously, this model is a single example system, and thus can not prove that the argument holds in general. Further study is needed to elucidate the relative advantages and disadvantages of interoceptive and exteroceptive behaviours (as well as how they might interact). Nevertheless, in addition to serving as an example for making the argument more explicit, analysis of the model demonstrates that even very simple forms of interoceptive behaviour can be remarkably robust to organisational-transformations.

Finally, we suggested that the adaptability of interoceptive behaviour can facilitate adaptive evolution and observed that interoceptive behaviour has already been produced in abiotic systems, and that its implementation could help in the synthesis of more robust artificial life.

## Additional Information

**How to cite this article**: Egbert, M. D. and Pérez-Mercader, J. Adapting to Adaptations: Behavioural Strategies That Are Robust to Mutations and Other Organisational-Transformations. *Sci. Rep.* **6**, 18963; doi: 10.1038/srep18963 (2016).

## Figures and Tables

**Table 1:** Reactions and equations used in the simulation of organisational-transformations that modify the autocatalytic reaction stoichiometry.

Pre-transformation reaction	Pre-transformation equation
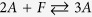	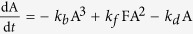
**Post-transformation reactions**	**Post-transformation equations**
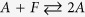	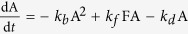
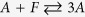	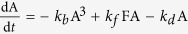
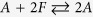	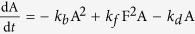
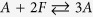	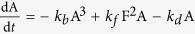
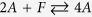	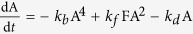
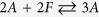	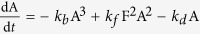
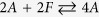	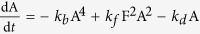
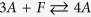	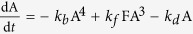
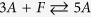	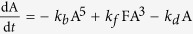
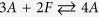	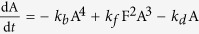
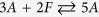	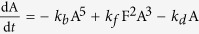
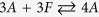	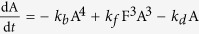
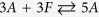